# Perceptions of social, emotional, and functional values in patients with type 2 diabetes mellitus and their satisfaction with primary health care services

**DOI:** 10.1017/S1463423619000471

**Published:** 2019-08-20

**Authors:** Aida Budrevičiūtė, Ramunė Kalėdienė, Liudmila Bagdonienė, Renata Paukštaitienė, Leonas Valius

**Affiliations:** 1 Department of Health Management, Faculty of Public Health, Medical Academy, Lithuanian University of Health Sciences, Kaunas, Lithuania; 2 Sustainable Management Research Group, School of Economics and Business, Kaunas University of Technology, Kaunas, Lithuania; 3 Department of Physics, Mathematics and Biophysics, Medical Academy, Lithuanian University of Health Sciences, Kaunas, Lithuania; 4 Department of Family Medicine, The Hospital of Lithuanian University of Health Sciences Kauno Klinikos, Kaunas, Lithuania

**Keywords:** competitive advantage, mixed methods, primary health care, satisfaction, type 2 diabetes mellitus, value perception

## Abstract

**Aim:**

To explore the relationships between social, emotional, and functional values, and satisfaction of patients with type 2 diabetes mellitus (T2DM) with an emphasis on Lithuanian primary health care services providers.

**Background:**

Academics and practitioners are encouraging more research on service value conceptually and empirically. Primary health care settings (PHCS) use modern management of value creation for patients with chronic diseases to increase the satisfaction of patients. Satisfaction of patients is the most important factor of competitive advantage for the PHCS. In this study, perceived value concept is dealt with in a multidimensional way. The fact that the perceived value in health sector has not previously been examined as multidimentionally has increased the importance of this research.

**Methods:**

The study strategy is based on focus group discussions of executives and survey of patients with T2DM in the primary health care sector. The target of focus group discussions is to gain knowledge about factors developing the competitive advantage of PHCS. The survey of patients with T2DM is the background to test the conceptual model of perceived value importance on satisfaction. The study uses coefficients of correlation, exploratory factor analysis, and linear regression.

**Findings:**

The results of focus groups revealed the factors of competitive advantage related to perspectives of health policy, organization, human resources, and patients. The results of the survey established statistically significant links between social value and satisfaction, and functional value and satisfaction. Emotional value decreased satisfaction of patients with T2DM.

## Introduction

Academics and practitioners are encouraging more research on service value conceptually and empirically, as value and services are inextricably linked (Gallarza *et al.*, 2017). A customer value-based theory of the organization highlights superior performance based on customer value organizational culture, learning customer’s changing needs, and innovations around customer value delivery process (Slater, 1997). Value for customers can be assessed not only on monetary effects but also on customer satisfaction, service quality, brand perception, length of customer relationships, customer base turnover, willingness to pay, employee satisfaction, and employee turnover (Grönroos, 2017). Scientists highlighted that the investigations were needed for how customers evaluate the gap between perceived and expected service (Zeithaml *et al.*, 1993). According to Holbrook (1996), the customer value is defined as an ‘interactive relativistic preference experience’ (Holbrook, 1996) and it is a complex construct of the specific satisfactions and dissatisfactions (Conti, 2013). The concept ‘perceived value’ emerged as an issue in the 1990s and has continued to receive extensive attention in the present century. The unidimensional approaches do not reflect the complexity of perceptions of value, whereas multidimensional constructs evidence holistic representations of phenomena (Sanchez-Fernandez and Iniesta-Bonillo, 2007). The various value dimensions on outcomes (satisfaction, peace of mind, financial quality of life, trust) may differ according to the length of the relationship with service provider (Plewa *et al.*, 2015). In the retail sector, perceived value dimensions included quality, emotional value, price, social value, and its recognition should enable retail managers to develop marketing positioning strategies (Sweeney and Soutar, 2001). When customers perceive to gain high emotional, social, and functional values from telecommunication operator’s innovation, they are more positive about consumption experience, purchase decision, and satisfaction (Mahmoud *et al.*, 2018). According to Chahal and Kumari (2012), customer perceived value in the health care sector comprises acquisition value, transaction value, efficiency value, esthetic value, social interaction value, and self-gratification value (Chahal and Kumari, 2012). Previous studies on value in preventative health described two value dimensions that are emotional and functional (Zainuddin *et al.*, 2013). Researchers proposed activities of value creation that included ensuring access and care continuity; developing a therapeutic relationship; providing evidence-based, planned diagnosis and treatment; and engaging patients through care planning (Rollow and Cucchiarra, 2016). The process of value assessment is the basis for a new theory development, including value strategies and expanding value for customer insights, increasing customer satisfaction and gaining a competitive advantage (Day and Crask, 2000). Scientists recognize that service value is multidimensional and there is no consensus about the number of types, classification criteria, or assessment (Gallarza *et al.*, 2017). There is increasing call by many scientists who are researching value and its dimensions to focus on customers’ satisfaction that enhance the competitive advantage of an organization. This study is opportune in that it strives to find answers to the following questions:

1. What are the factors for developing the competitive advantage of primary health care settings (PHCS)?
2. What are the relationships between perception of emotional, social, and functional values, and sociodemographic characteristics of patients with type 2 diabetes mellitus (T2DM)?
3. What are the relationships between emotional, social, and functional values, and satisfaction of patients with T2DM?
4. What is the growth of competitive advantage opportunities of PHCS?

## Theoretical foundation and hypothesis development

Consumer focus groups were conducted to explore concerns, knowledge, and beliefs around prevention of T2DM, and the study results found out that the most important areas are risk factors, nature of condition, and preventative benefit of lifestyle changes (Berryman *et al.*, 2009). The qualitative study executed with patients diagnosed with diabetes and having poor glycemic control identified a wide variety of strategies on how to manage their disease (Berenguera *et al.*, 2016). Self-management is the best strategy of disease management, but is often difficult due to family or economic reasons, a lack of awareness, or a lack of motivation (Berenguera *et al.*, 2016). A longitudinal study (*n* = 26344) of patients with T2DM conducted in Australia found that diabetes has important impacts on quality of life, social contacts, and, as a result, it may have negative effects on mental health and T2DM management in the long-term perspective (Feng and Astell-Burt, 2017). A participatory research with patients (*n* = 79) living with diabetes expressed concern regarding accessibility, organization, coordination, better dissemination, and visibility of services (Vachon *et al.*, 2017). The study (*n* = 192) of collaborative goals (listen and learn, share ideas, caring relationship, agree on objective, support) between doctor and patients with diabetes showed there to be significant association with increased perceived self-management competence, which was significantly associated with increased self-management behaviors (Morris *et al.*, 2017). In Saudi Arabia, the results of the study (*n* = 383) showed that diabetic patients need more education programs about how to identify and manage symptoms of disease (Al-Ghamdi *et al.*, 2018).

Oliver (1980) expressed that satisfaction is the function of expectation and expectancy disconfirmation, and satisfaction influences attitude change and intention of purchase (Oliver, 1980) and consumers will be satisfied when their assessment of a service or product confirms their expectations (Roig *et al.*, 2013). The measures of consumer perceptions of health care services, as well as satisfaction, include such enabling components as the continuity of care, availability and convenience of services, and various access mechanisms (cost, payment, ease of emergency care facilities) (Ware *et al.*, 1975). The major patient satisfaction theories were published in the 1980s and five key theories can be identified: Discrepancy and transgression theories of Fox and Storms (1981); Expectancy-value theory by Linder-Pelz (1982); Determinants and components theory of Ware *et al.* (1983); Multiple models theory of Fitzpatrick and Hopkins (1983); and Health care quality theory of Donabedian (1980) (Gill and White, 2009).

The emotional service experience and the feelings generate a positive attitude toward the brand (Roig *et al.*, 2013). Emotional benefits in community-supported agriculture refer to the perceptual benefits acquired from feelings and/or affective states (Chen, 2013). In general, emotional value is described by the utility that is derived from the feelings or affective states that a product or service generates (Sweeney and Soutar, 2001). In personal health, emotional value can refer to the promotion of positive or negative emotions (Zainuddin *et al.*, 2013). In literature, social value is named as the utility that derived from the product or service ability to enhance social self-concept (Sweeney and Soutar, 2001). Social value is important in order to make the final decision, in that sense, it is necessary for any financial institution to maintain a good social reputation (Roig *et al.*, 2013). In community-supported agriculture, social benefits are perceptual benefits acquired by association with social class and social status (Chen, 2013). Functional value focuses on performance, functionality, and include economic benefit or the utility that is provided by a product or service (Zainuddin *et al.*, 2013).

Competition in health care involves elements such as price, quality, convenience, superior products and/or services, new technology, and innovation (Rivers and Glover, 2008). Studies show that competition is capable of increasing value for customers and satisfied patients are more likely to better comply with providers medical treatment and to cooperate or maintain relationships with providers (Rivers and Glover, 2008). The competitive advantage is unique position of organization (Porter, 1991) that can be gained through resources (Barney, 1991) and effective management of people (Pfeffer *et al.*, 1995). Analysis of the chain of the effects value–satisfaction–loyalty revealed the strong link between perceived value and customer satisfaction (Gallarza *et al.*, 2016) and this link can strenghten long-term relationship with customers and to achieve a competitive advantage (Chahal and Kumari, 2012).

**Figure 1.**
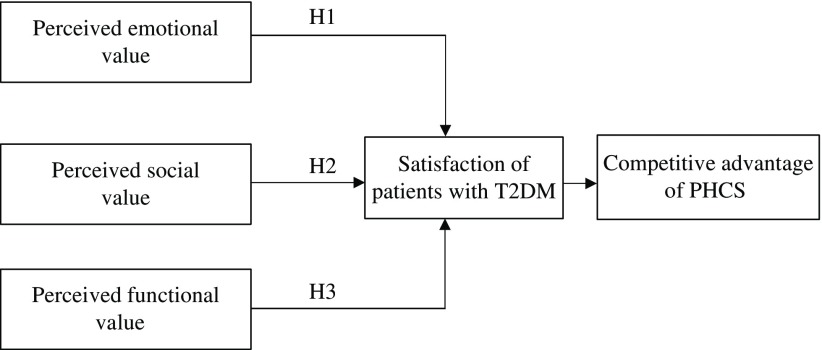
Proposed model of value perception importance on satisfaction

**Source:** researchers’ own (2017).

From the scientific discussion, the researchers propose a conceptual model presented in Figure 1. The proposed model theorizes that competitive advantage of PHCS is enhanced by increasing the satisfaction of patients during value creation process.

Our study objectives are:

1. to determine the factors to develop the competitive advantage of PHCS in the point of view of executive managers of PHCS;
2. to investigate the conceptualization of customer perceived value in the context of primary health care services;
3. to develop a conceptual model of the impact of perceived value dimensions on patients with T2DM satisfaction; and
4. to explore the value dimension perceptions of patients with T2DM in relevance to satisfaction after the consultation of family doctor in PHCS.

## Statistical methods

The construct validity of the questionnaire was tested with exploratory factor analysis and reliability of the questionnaire was tested using Cronbach’s alpha. The scores of the factor analysis were analyzed as estimates of the emotional, social, functional, and satisfaction values. Spearman’s rank correlation coefficient (*r*_s_) was used to analyze the linear relation between factor scores and quantitative features. Linear regression analysis was used for modeling the relationship between the satisfaction of respondents and their emotional, social, and functional values. For the analysis of the relation between factor scores and qualitative features, factor scores were grouped into two groups: weakly (factor score less or equal to zero) and strongly (factor score bigger than zero) expressed emotional, social, functional, and satisfaction values. Cramer's coefficient (*r*_cr_) and Chi-square test for independence were used to analyze the relation between qualitative features. Observed correlation was assumed as statistically significant if *P* value <0.05.

## Material and methods

**Figure 2.**
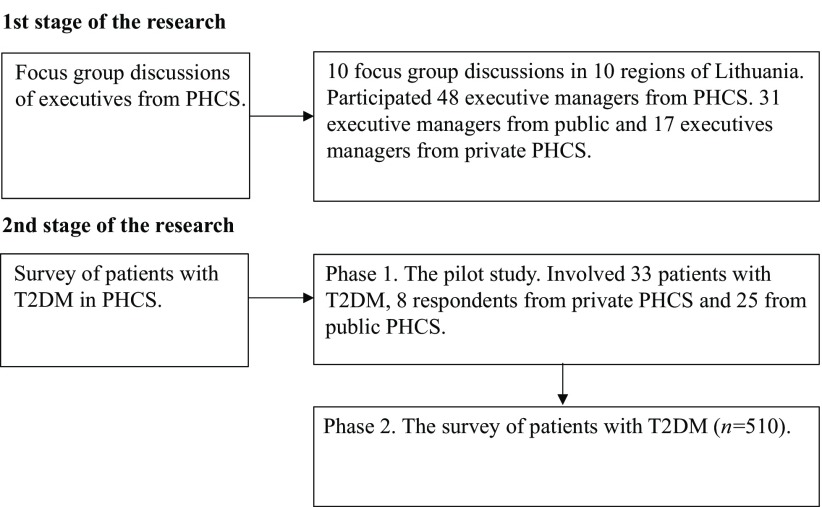
The research design

**Source:** researchers’ own (2015).

The present study conceptualizes competitive advantage and customer perceived value in the context of primary health care services. The study is based on the analysis of scientific literature, focus group discussions of executives of PHCS, and survey of patients with T2DM in PHCS. The research design is presented in Figure 2.

The qualitative study is based on the opinion of the participants of focus group discussions toward factors of competitive advantage of the PHCS. Focus group discussions were conducted from May 2015 to March 2016 in 10 counties of Lithuania. A total of 48 participants were enrolled into the qualitative study: 31 leaders of public PHCS and 17 leaders of private primary health care organizations. The mean size of the focus group was five participants. The mean duration of the focus group discussion was 1.21 h. Participants were selected from the list composed by the Lithuanian Institute of Hygiene at the end of 2012. Selection was done following the principle of 50/50, with the intention to include the executives of both public and private PHCS. The executive of the PHCS was contacted by phone and informed about the topic, purpose, time, and place of focus group discussion. If the leader of the PHCS agreed to participate in the focus group session, an invitation was sent by email and/or presented to the participant or to hospital staff, which gave the invitation to the executive. Informed consent to participate in focus group sessions was obtained. Focus group sessions were audiotaped and based on the records, analysis of focus group discussions was performed. In order to assess the quality of focus group discussions, the questionnaires were sent to participants of focus group discussions by email after the study. The second stage of the research is a survey of patients with T2DM after a consultation with family doctor in PHCS in Lithuania. The study's object is opinion of patients with T2DM about value creation management and their satisfaction with primary health care services. The questionnaire was built following a methodological process based on information sources that included consultations with scientists, results of focus group discussions, and scientific literature review. Indicators and sources for the study variables are provided in Table 1.

**Table 1.** Indicators and sources for the study variables

Variable	Source	Construct	Code
Emotional value	Feng and Astell-Burt (2017) and Al-Ghamdi *et al.* (2018)	Diabetes affects my feelings	EMV1
	Al-Ghamdi *et al.* (2018)	Diabetes raises for me many worries	EMV2
	Feng and Astell-Burt (2017) and Al-Ghamdi *et al.* (2018)	Diabetes worsen my life quality	EMV3
Social value	Alotaibi *et al.* (2015)	Given services of my family doctor improves understanding about health	SOV1
	Alotaibi *et al.* (2015)	I appreciate communication with my family doctor	SOV2
	Consultations with scientists	I appreciate communication with nurse	SOV3
Functional value	Vachon *et al.* (2017)	Given service of my family doctor fulfilled my expectations	FUV1
	Consultations with scientists, focus group discussions	Given service of nurse fulfilled my expectations	FUV2
	Alotaibi *et al.* (2015)	Given service of reception personnel fulfilled my expectations	FUV3
	Lim and Tang (2000); Bakar *et al.* (2008); Chahal and Kumari (2012)	Cleanliness of family health care institution fulfilled my expectations	FUV4
	Lim and Tang (2000); Bakar *et al.* (2008)	Working time of family health care institution fulfilled my expectations	FUV5
	Roig *et al.* (2013)	Diagnostic examination in family health care setting fulfilled my expectations	FUV6
	Consultations with scientists, focus group discussions	In family health care setting, I got information about health promotion programs	FUV7
Customer satisfaction	Consultations with scientists, focus group discussions	I am satisfied with given service of family doctor	CUS1
	Consultations with scientists, focus group discussions	I am satisfied with given service of nurse	CUS2
	Consultations with scientists, focus group discussions	I am satisfied with given service of reception personnel	CUS3
	Malik *et al.* (2016)	I favorably respond to others about PHCS	CUS4
	Consultations with scientists, focus group discussions	I am satisfied with diabetes treatment at this PHCS	CUS5

PHCS = primary health care settings.

In phase 2, a pilot study was conducted in May 2017 in Lithuania to evaluate the suitability of questionnaire. The executives of the PHCS were informed by phone and/or by email about the pilot study. The personal information and informed consent forms and questionnaires were distributed to executives or heads of departments or family doctors working in PHCS. The questionnaires were filled out by patients with T2DM after a consultation with family doctor. The pilot study involved 33 patients with T2DM from private PHCS (8 respondents) and from public PHCS (25 respondents). In total, 80 questionnaires were distributed, 33 questionnaires were completed (41% response rate). The reliability of questionnaire was evaluated by Cronbach’s α that was 0.920. Taking into account the opinion of respondents, the items of questionnaire were corrected and developed. During the period October 2017–January 2018, the survey was conducted, and data were collected using the questionnaire for patients with T2DM in private and in public PHCS in Lithuania. In total, 701 respondents were approached to take part in the survey and 510 valid questionnaires (258 from public and 252 from PHCS) were obtained, resulting in a 72.8% response rate.

## Results of the study

During the focus group discussions, the researchers sought to puzzle out the factors developing the competitive advantage of PHCS. The factors of competitive advantage are presented in Table 2.

**Table 2.** Factors of competitive advantage of public and private PHCS

	Factors of competitive advantage	
Perspectives	Public PHCS	Private PHCS
Health policy	Burnout	Law
	Psychology	Competition
	Media	Services
	Law	
Organization	Infrastructure	Image
	Budget	Infrastructure
	Place	Place
	Patients	Quality
	Initiatives	
Human resources	Competencies	Teamwork
	Motivation	Partnership
	Workload	Satisfaction
		Results
		Communication
		Prestige
Patients	Communication	Culture
	Loyalty	Quality
	Relationships	Contacts
	Choice	Satisfaction
	Power	
	Satisfaction	

PHCS = primary health care settings.

During the focus group discussions, the executives from PHCS expressed that the competition in primary health care sector is needed and this phenomenon can provide for patients to choose between alternatives. Most often the patient evaluates the family doctor's given service by communication, not by quality. On the contrary, the leaders from private PHCS point out the service quality and culture. In public PHCS, the competitive advantage is created through communication between patient and family doctor and patient’s loyalty to family doctor. Value of family doctor’s work and profession prestige in society are the main strategic benchmarks of human resources of private PHCS. Staff competencies, motivation, and workload are the priorities of human resources management of public PHCS.

In the second phase of the study, the survey was completed. The sample profile of the survey is provided in Table 3.

**Table 3.** Sample profile

Respondents characteristics		*n*	%
Gender	Female	350	68.6
	Male	160	31.4
Residence	Urban	406	79.6
	Rural	104	20.4
Education	Primary	91	17.8
	Secondary	204	40
	Higher college	116	22.7
	Higher university	99	19.4
Income	Less than 350 eur	313	61.4
	Over 350 eur	197	38.6
Occupation status	Retired	183	35.9
	Employees of physical work	147	28.8
	Employees of intellectual work	163	32.0
	Other (housewife, unemployed)	17	3.3
Affiliation to PHCS	Private	252	49.4
	Public	258	50.6

PHCS = primary health care settings.

Factor analysis employed to explore emotional, social, and functional values and satisfaction components among Lithuanian primary health care services providers. In total, two items were eliminated: emotional value and social value, because they did not contribute to a simple factor structure and failed to meet the loading factor criteria (meaning 0.5 or above). The scale’s internal consistency examined using Cronbach’s α and each construct are within expected range. Each factor name is based on the variables with significant loadings (Cronbach’s α, Kaiser–Meyer–Olkin measure (KMO)):

- Factor 1, emotional value, 3 items, Cronbach’s α 0.838, KMO 0.704.
- Factor 2, social value, 3 items, Cronbach’s α 0.851, KMO 0.659.
- Factor 3, functional value, 7 items, Cronbach’s α 0.937, KMO 0.915.
- Factor 4, satisfaction, 5 items, Cronbach’s α 0.935, KMO 0.856.

The factor and Cronbach’s α analysis are shown in Table 4.

**Table 4.** Results of exploratory factor analysis and Cronbach’s α analysis

	Factor analysis			Cronbach’s α analysis	
Factor name	Constructs	Loadings	Variance explained	Corrected item, total correlation	Alpha if deleted
Emotional value	EMV1	0.848		0.663	0.813
	EMV2	0.905	75.832	0.767	0.715
	EMV3	0.858		0.679	0.798
Social value	SOV1	0.802		0.605	0.899
	SOV2	0.934	77.401	0.825	0.693
	SOV3	0.898		0.745	0.769
Functional value	FUV1	0.872		0.819	0.925
	FUV2	0.900		0.854	0.922
	FUV3	0.780		0.705	0.936
	FUV4	0.912	73.223	0.871	0.921
	FUV5	0.876		0.825	0.925
	FUV6	0.858		0.802	0.927
	FUV7	0.781		0.709	0.937
Satisfaction	CUS1	0.911		0.852	0.916
	CUS2	0.907		0.847	0.917
	CUS3	0.865	79.659	0.790	0.928
	CUS4	0.897		0.838	0.918
	CUS5	0.882		0.816	0.922

Statistical data analysis established statistically significant correlation between patients with T2DM age and emotional value (*r*_s_ = 0.144, *P* < 0.001), and between patients with T2DM age and satisfaction value (*r*_s_ = 0.115, *P* = 0.010). The statistically significant correlation is estimated between disease treatment duration and functional value (*r*_s_ = 0.115, *P* = 0.009), emotional value (*r*_s_ = 0.162, *P* < 0.001), and satisfaction value (*r*_s_ = 0.104, *P* = 0.019). The statistical data analysis of grouped (weakly and strongly expressed) emotional, social, functional, and satisfaction data showed which sociodemographic characteristics has statistically significant influence on analyzed characteristics. Residence (*r*_cr_ = 0.151; *P* < 0.001), affiliation to PHCS (*r*_cr_ = 0.129; *P* = 0.0041), and occupation (*r*_cr_ = 0.13; *P* = 0.034) of respondents have statistically significant influence on grouped emotional value (Table 5).

**Table 5.** Perception of emotional value by sociodemographic characteristics

		Emotional value			
Characteristics		Weakly expressed, *n* (%)	Strongly expressed, *n* (%)	Cramer’s coefficient	*P-value
Gender	Female	164 (68.9)	186 (68.4)	0.006	0.899
	Male	74 (31.1)	86 (31.6)		
Residence	Urban	174 (73.1)	232 (85.3)	0.151	0.001
	Rural	64 (26.9)	40 (14.7)		
Income	≤350 eur	151 (63.4)	162 (59.6)	0.04	0.368
	>350 eur	87 (36.6)	110 (40.4)		
Education	Primary	54 (22.7)	37 (13.6)	0.123	0.052
	Secondary	90 (37.8)	114 (41.9)		
	Higher college	48 (20.2)	68 (25)		
	Higher university	46 (19.3)	53 (19.5)		
Affiliation to PHCS	Private	134 (56.3)	118 (43.4)	0.129	0.004
	Public	104 (43.7)	154 (56.6)		
Occupation	Retired	74 (31.1)	109 (40.1)	0.13	0.034
	Employees of physical work	82 (34.5)	65 (23.9)		
	Employees of intellectual work	76 (31.9)	87 (32)		
	Other (housewife, unemployed)	6 (2.5)	11 (4)		

* Pearson Chi-square test for independence, data are given as *n* (%).

PHCS = primary health care settings.

Income of respondents (*r*_cr_ = 0.098; *P* = 0.026) and occupation of respondents (*r*_cr_ = 0.145; *P* = 0.013) had statistically significant influence on grouped functional value (Table 6).

**Table 6.** Perception of functional value by sociodemographic characteristics

		Functional value			
Characteristics		Weakly expressed, *n* (%)	Strongly expressed, *n* (%)	Cramer’s coefficient	*P-value
Gender	Female	136 (72.7)	214 (66.3)	0.067	0.129
	Male	51 (27.3)	109 (33.7)		
Residence	Urban	148 (79.1)	258 (79.9)	0.009	0.843
	Rural	39 (20.9)	65 (20.1)		
Income	≤350 eur	103 (55.1)	210 (65)	0.098	0.026
	>350 eur	84 (44.9)	113 (35)		
Education	Primary	31 (16.6)	60 (18.6)	0.058	0.633
	Secondary	80 (42.8)	124 (38.4)		
	Higher college	44 (23.5)	72 (22.3)		
	Higher university	32 (17.1)	67 (20.7)		
Affiliation to PHCS	Private	91 (48.7)	161 (49.8)	0.011	0.797
	Public	96 (51.3)	162 (50.2)		
Occupation	Retired	62 (33.2)	121 (37.5)	0.145	0.013
	Employees of physical work	48 (25.7)	99 (30.7)		
	Employees of intellectual work	65 (34.8)	98 (30.3)		
	Other (housewife, unemployed)	12 (6.4)	5 (1.5)		

* Pearson Chi-square test for independence, data are given as *n* (%).

PHCS = primary health care settings.

Grouped social value did not depend on any sociodemographic characteristics. Table 7 presents the perception of social value by sociodemographic characteristics.

**Table 7.** Perception of social value by sociodemographic characteristics

		Social value			
Characteristics		Weakly expressed, *n* (%)	Strongly expressed, *n* (%)	Cramer’s coefficient	*P-value
Gender	Female	89 (70.6)	261 (68)	0.025	0.576
	Male	37 (29.4)	123 (32)		
Residence	Urban	101 (80.2)	305 (79.4)	0.008	0.86
	Region	25 (19.8)	79 (20.6)		
Income	≤350 eur	75 (59.5)	238 (62)	0.022	0.623
	>350 eur	51 (40.5)	146 (38)		
Education	Primary	20 (15.9)	71 (18.5)	0.034	0.901
	Secondary	53 (42.1)	151 (39.3)		
	Higher college	28 (22.2)	88 (22.9)		
	Higher university	25 (19.8)	74 (19.3)		
Affiliation to PHCS	Private	67 (53.2)	185 (48.2)	0.043	0.33
	Public	59 (46.8)	199 (51.8)		
Occupation	Retired	45 (35.7)	138 (35.9)	0.03	0.928
	Employees of physical work	34 (27)	113 (29.4)		
	Employees of intellectual work	43 (34.1)	120 (31.3)		
	Other (housewife, unemployed)	4 (3.2)	13 (3.4)		

* Pearson Chi-square test for independence, data are given as *n* (%).

PHCS = primary health care settings.

In Table 8, the satisfaction by sociodemographic characteristics is presented. Only income of respondents had weak but statistically significant influence on grouped value of satisfaction (*r*_cr_ = 0.09, *P* = 0.043).

**Table 8.** Satisfaction of respondents by sociodemographic characteristics

		Satisfaction value			
Characteristics		Weakly expressed, *n* (%)	Strongly expressed, *n* (%)	Cramer’s coefficient	*P-value
Gender	Female	105 (75)	245 (66.2)	0.084	0.056
	Male	35 (25)	125 (33.8)		
Residence	Urban	116 (82.9)	290 (78.4)	0.05	0.263
	Rural	24 (17.1)	80 (21.6)		
Income	≤350 eur	76 (54.3)	237 (64.1)	0.09	0.043
	>350 eur	64 (45.7)	133 (35.9)		
Education	Primary	23 (16.4)	68 (18.4)	0.106	0.125
	Secondary	57 (40.7)	147 (39.7)		
	Higher college	40 (28.6)	76 (20.5)		
	Higher university	20 (14.3)	79 (21.4)		
Affiliation to PHCS	Private	67 (47.9)	185 (50)	0.019	0.666
	Public	73 (52.1)	185 (50)		
Occupation	Retired	52 (37.1)	131 (35.4)	0.08	0.352
	Employees of physical work	33 (23.6)	114 (30.8)		
	Employees of intellectual work	51 (36.4)	112 (30.3)		
	Other (housewife, unemployed)	4 (2.9)	13 (3.5)		

* Pearson Chi-square test for independence, data are given as *n* (%).

PHCS = primary health care settings.

The findings showed that the stongest linear association was between satisfaction and functional value (*r*_sp_ = 0.603, *P* < 0.001), and between satisfaction and social value (*r*_sp_ = 0.598, *P* < 0.001). Furthermore, emotional value showed weak dependence with all remaining values (Table 9).

**Table 9.** Spearman’s rank coefficient matrix for all analyzed values

Characteristics	Satisfaction value	Functional value	Social value	Emotional value
Satisfaction value	–	0.603 (*P* < 0.001)	0.598 (*P* < 0.001)	0.149 (*P* < 0.001)
Functional value	0.149 (*P* < 0.001)	–	0.501 (*P* < 0.001)	0.193 (*P* < 0.001)
Social value	0.598 (*P* < 0.001)	0.501 (*P* < 0.001)	–	0.282 (*P* < 0.001)
Emotional value	0.149 (*P* < 0.001)	0.193 (*P* < 0.001)	0.282 (*P* < 0.001)	–

The coefficients of linear regression were used to support the research hypothesis about satisfaction value (determination coefficient 0.687). Only the research hypothesis H1 was not supported because coefficient of linear regression showed negative effect of emotional value on satisfaction value (increased emotional value will decrease average satisfaction value). H2 and H3 research hypotheses were supported (Table 10).

**Table 10.** The research hypothesis testing

				95% confidence interval for β		
Hypothesis	*β*	*t*	*P*	Lower bound	Upper bound	Hypothesis testing results
H1: Patients with T2DM perceived emotional value is directly and positively related to satisfaction.	−0.074	−2.783	0.006	−0.127	−0.022	Not supported
H2: Patients with T2DM perceived social value is directly and positively related to satisfaction.	0.579	15.442	<0.001	0.506	0.653	Supported
H3: Patients with T2DM perceived functional value is directly and positively related to satisfaction.	0.331	9.100	<0.001	0.260	0.403	Supported

T2DM = type 2 diabetes mellitus.

## Discussion

Customers’ expectations are important for value perception, satisfaction, and behavior intentions in value creation processes. In Singapore hospitals, a generic, internationally used market research technique SERVQUAL developed by Parasuraman *et al.* (1985) was used to determine the expectations and perceptions of patients (*n* = 252) (Lim and Tang, 2000). The study results found that patients had the highest expectations in the assurance, reliability, and responsiveness dimensions (Lim and Tang, 2000). In Turkish hospitals, patients (*n* = 472) expressed the lowest service scores in responsiveness and reliability dimensions (Bakar *et al.*, 2008). During the focus group discussions, the managers from public PHCS expressed that the main elements of patients’ perspective are communication, loyalty, relationships, choice, power, and satisfaction. The managers from private PHCS mentioned that the sources of competitive advantage are culture, quality, contacts, and satisfaction. The essential element of patients’ perspective is their satisfaction with given services of family doctor, nurse, reception personnel, and satisfaction with disease treatment. The Indian study (*n* = 100) findings revealed that 83.5% of the patients were satisfied with the general experience and the behavior of the health care provider, 85.9% were satisfied with the treatment, and 65.5% were satisfied with physical environment of the clinic (Ardey and Ardey, 2015). The main objective of our study was to explore the way value dimension (emotional, functional, social) perceptions of patients with T2DM vary in relevance to satisfaction after the consultation of family doctor in PHCS. It is proven by the Malaysian study (*n* = 170) in the restaurant business industry that customer satisfaction influenced by perceived value and monetary price is seen to be the best predictor, and the results indicate that emotional response effects customer satisfaction (Raji and Zainal, 2016). Other dimensions as behavioral price and reputation also contribute to customer satisfaction (Raji and Zainal, 2016). In Kuwait, primary health care services were evaluated and were found statistically significant differences of patients‘ satisfaction with gender and their education (Alotaibi *et al.*, 2015). In Canada, during the research of patients (*n* = 252), using complex continuing care and rehabilitation services, satisfaction was assessed and it was found that gender made a significant contribution to overall satisfaction (Malik *et al.*, 2016). In our study, it was found that if emotional value increases per 1 unit, average of patients’ satisfaction decreases by 0.074. In contrast, it is demonstrated that the perceived value of emotional benefits has a direct and positive effect on customer satisfaction in the banking sector (Roig *et al.*, 2013). In our study, the hypothesis that patients with T2DM perceived functional value are directly and positively related to satisfaction was confirmed. The banking sector also confirmed that the perceived value of functional benefits has a direct and positive effect on customer satisfaction (Roig *et al.*, 2013). Our study found that patients with T2DM perceived social value have the highest effect on patients’ satisfaction. The results of other studies showed that the value of functional benefits is the variable that contributes the highest value to overall satisfaction and is the most influential factor in the final decision to return (Roig *et al.*, 2013). This research advances understanding of the concept of perceived value and its dimensions (emotional, social, functional) in the primary health care sector. In this study, we propose factors developing the competitive advantage of the PHCS, a model of perceived value on satisfaction in primary health care sector, define its constructs, develop measures of these constructs, test their validity and the reliability of the measures, and examine the influence perceived value of patients with T2DM on their satisfaction in the context of competitive advantage of PHCS.

## Conclusions

The research findings from this study contribute to marketing of health services theory and extend the insights into disease management by showing the influence of value dimensions on the satisfaction of patients with T2DM. During the health care services delivery, the value for patients is created using the resources of the PHCS. Satisfaction of patients is the most important factor for developing the competitive advantage of PHCS. The perception of value importance associates with sociodemographic characteristics of patients with T2DM (duration of disease treatment, age, residence, gender, income, and occupation). Emotional value showed a negative influence on patients’ satisfaction, while social and functional values showed a significant positive influence on patients’ satisfaction. The growth opportunities of competitive advantage of PHCS sourced from the results of this study:

- to develop value management strategies increasing the satisfaction of patients with T2DM;
- to build marketing programs on the strength of sociodemographics variables of patients with T2DM; and
- to bring up the satisfaction of family doctors, nurses, and other front-line staff of PHCS.

## Limitations and directions for future research

As with any scientific research, there are number of study limitations and future directions. Researchers pointed out that the strategy of study is adapted for chronic disease in the context of services of primary health care. It is the case to compare the conceptual model within other health care services, other diseases, and to find similarities/dissimilarities. The results of the study showed the perception of value dimensions of patients with chronic disease in Lithuanian primary health care sector and it could be the direction to compare it with other countries. The study involved the managers from PHCS and it would be interesting to research the opinion of patients about priorities of primary health care. It is valuable to evaluate opinion of patients about what they valued in primary health care services.
